# Targeting parallel bypass signaling to combat adaptive resistance to BRAF inhibition in colorectal cancer

**DOI:** 10.18632/oncoscience.401

**Published:** 2018-04-29

**Authors:** Chenxi Gao, Jing Hu

**Affiliations:** Department of Pharmacology and Chemical Biology, UPMC Hillman Cancer Center, University of Pittsburgh School of Medicine, Pittsburgh, PA 15213, USA

**Keywords:** BRAF, CRC, resistance, the Wnt/b-catenin pathway, FAK

BRAF is a serine/threonine kinase that is downstream of KRAS and immediate upstream of MEK (mitogen-activated protein kinase kinase), and about 8-15% of all colorectal cancer (CRC) patients harbor activating mutations (mostly V600E mutation) of BRAF. *BRAF*-mutated CRC is a distinct molecular phenotypic and clinical subset that does not respond to chemotherapy, anti-EGFR (Epidermal Growth Factor Receptor) therapy and had the highest mortality. One of the most encouraging developments in oncology has been the success of BRAF inhibitors in *BRAF*-mutant melanoma: the initial response rate in melanoma is 60-80% [1]. However, BRAF inhibitor monotherapy is ineffective with 0-5% response rate in *BRAF*-mutant CRCs [2]. Tremendous effort has been put forth to develop effective strategies to overcome BRAF resistance, but thwarting resistance to BRAF inhibitors remains one of the major clinical challenges in therapy against CRC. New perspective can lead to new solution.

Many studies on BRAF inhibitor resistance in CRC have focused on mechanisms underlying the adaptive reactivation of the EGFR/RAS/RAF/MEK/ERK pathway. Indeed, triple inhibition of EGFR/BRAF/MEK showed better results and patients response rate was improved to 21% [3]. However, comparing to patients response rate in melanoma, there's still large room to improve. We propose that effective and durable control of BRAF- mutated CRC will unlikely be achieved via vertical pathway targeting only. CRC-specific pathway bypass mechanisms also need to be taken into consideration. We therefore approached the BRAF resistance problem from a previously unexplored angle: identifying BRAF inhibitor treatment-triggered molecular events that cause opposing functional outcomes in melanoma cells vs. in CRC cells. And we have identified Wnt/β-catenin pathway activation as such an event [4].

Wnt/β-catenin signaling functions as a tumor suppressor in melanoma biology [5] and activation of the Wnt pathway is essential for the anti-tumor effects of BRAF inhibitors [6]; however, in CRC, it is a critical driving force behind tumor initiation and development [7]. We recently reported that FAK stabilizes β-catenin by phosphorylating glycogen synthase kinase 3α/β at tyrosine 279/216 (GSK3α/βY279/Y216) [8]. We found that treatment with BRAF inhibitors (both current and next generation BRAF inhibitors) upregulated the Wnt/β-catenin pathway in BRAFV600E-mutant CRC cells through FAK activation-mediated phosphorylation of GSK3α/ βY279/Y216 [4], an event that was independent of EGFR or ERK1/2 (extracellular-signal-regulated kinases1/2) activation, indicating that BRAF inhibitor treatment- induced hyperactivation of Wnt signaling is “pathway reactivation”-independent. Co-targeting of BRAF/ Wnt pathways or BRAF/FAK pathways exerted strong synergistic antitumor effects in cell culture and mouse xenograft models. Overall, our study suggests a novel “bypass” signaling mechanism: BRAF inhibition-induced hyperactivation of the Wnt/β-catenin pathway, which is independent of MAPK pathway reactivation. Given Wnt pathway's unequivocal role in CRC, we believe Wnt/β- catenin pathway activation is one of the root causes of CRC resistance to BRAF inhibition. Our results suggest that while complete vertical pathway blockade is pivotal for effective and durable control of *BRAF*-mutant CRC, co-targeting parallel adaptive signaling—the Wnt/β- catenin pathway—is also essential. Wnt/β-catenin signaling pathway has been difficult to target and few specific Wnt pathway inhibitors have just entered clinical trials. Our study offered a feasible solution for targeting the Wnt pathway activation: pharmacological inhibition of FAK.

The next generation of RAF inhibitors has recently been developed. These new inhibitors possess much more potent MAPK inhibition activities and may provide enhanced safety and efficacy over first- generation of BRAF inhibitors. We found that treatment with LY3009120 (a new Pan-RAF inhibitor) or PLX7904 (a new paradox-breaking RAF inhibitor) activated FAK and induced β-catenin accumulation similarly as the current generation of BRAF inhibitors without inducing ERK reactivation. Again, co-targeting of Wnt pathway activation using a Wnt pathway inhibitor or FAK inhibitor exerted strong synergistic inhibitory effects with these next generation BRAF inhibitors. Our findings led us to predict that *BRAF*-mutant colorectal tumors will exhibit similar levels of resistance to second generation BRAF inhibitors as to current inhibitors and the next generation of BRAF inhibitor as monotherapy will be ineffective in the treatment of *BRAF*-mutant CRC patients. That said, we suggest that the same strategy can be used to overcome colon cancer resistance to both generations of BRAF inhibitors.

Taken together, our recent study has identified a novel parallel bypass mechanism—FAK-dependent Wnt/ β-catenin pathway hyperactivation—as a potential root cause for BRAF inhibitor resistance in CRC. In light of previous findings in melanoma [5, 6], we propose that the Wnt/β-catenin pathway activation may explain at least partly why the initial patient response rates are drastically different in *BRAF*-mutated melanomas and CRC. Co- targeting the Wnt/β-catenin pathway using FAK inhibitors may represent a new feasible solution to overcome resistance to both generations of BRAF inhibitors with great potential to dramatically change the outlook for *BRAF*-mutated CRC patients.
